# Cardiac glycoside bufalin blocks cancer cell growth by inhibition of Aurora A and Aurora B activation via PI3K-Akt pathway

**DOI:** 10.18632/oncotarget.24475

**Published:** 2018-02-09

**Authors:** Chuan-Ming Xie, Xiao-Tong Lin, Di Wu, Ye Tan, Christopher H.K. Cheng, Jun Zhang

**Affiliations:** ^1^ Institute of Hepatobiliary Surgery, Southwest Hospital, Third Military Medical University (Army Medical University), Chongqing, China; ^2^ School of Biomedical Sciences, The Chinese University of Hong Kong, Shatin, New Territories, Hong Kong, China; ^3^ Center of Novel Functional Molecules, The Chinese University of Hong Kong, Shatin, New Territories, Hong Kong, China; ^4^ Department of Hematopathology, University of Texas MD Anderson Cancer Center, Houston, TX, USA

**Keywords:** bufalin, Aurora kinase, PI3K/Akt, cancer, mitosis

## Abstract

In our previous study, cardiac glycosides including bufalin, a group of sodium pump (Na+/K+-ATPase) inhibitors widely used to treat heart failure for many years, have been demonstrated to induce a delay of mitotic entry and mitotic arrest in many cancer cells. However, the underlying mechanism remains poorly understood. Here, we reported for the first time that cardiac glycoside bufalin induced mitotic entry delay and prometaphase arrest by inhibition of activation of Aurora A/B. Furthermore, cardiac glycoside bufalin prevented Aurora A recruitment to mitotic centrosomes and Aurora B recruitment to unattached kinetochores. Mechanistically, bufalin and knockdown of sodium pump inhibited PI3K-Akt pathway, which in turn inhibit the activation of Aurora A/B, followed by a delay in mitotic entry and mitotic arrest. These actions were reversed by overexpression of Akt. In addition, ERK, mTOR, and ROS are not involved in bufalin-mediated downregulation of active form of Aurora A/B. Taken together, cardiac glycoside bufalin induces mitotic entry delay and mitotic arrest in cancer cells through inhibition of Aurora A/B activation via PI3K-Akt pathway. Based on this novel finding we could suggest that targeting PI3K-Akt pathway may have therapeutic value for the treatment of cancers associated with sodium pump overexpression.

## INTRODUCTION

The sodium pump is one of the most ubiquitously expressed proteins at the plasma membrane of all mammalian cells, which is known for its critical role in regulating cell volume and membrane potential by transporting 3 Na+ out and 2 K+ into cells utilizing energy from ATP hydrolysis. In the past decades, studies have reported that sodium pump functions as a signaling transducer that regulates Src kinase, NF-κB and PI3K [1–3]. Sodium pump is a heterodimer of two subunits, the α catalytic subunit and the β subunit, together with a regulatory γ subunit (FXYD), and each type of subunit has its isoforms [4, 5]. A functional unit of sodium pump would comprise minimally one α- and one β-subunit [5]. There are four isoforms of α (catalytic) subunit (α1-4) and three isoforms of β (β1-3). The α1β1 complex is the ubiquitous complex; α2 in heart and skeletal muscle; α3 in the brain; and α4 in the testis and spermatozoids. Since the award of a Nobel Prize in 1997 to Jens Christian Skou for his first discovery of the sodium pump in 1957 [6], many researchers have investigated into the structure and functions of the sodium pump. Besides regulating cell volume and membrane potential, the sodium pump has been reported to play a key role in cell growth, differentiation and cell death, but the mechanisms are still not completely understood.

Cardiac glycosides, a serial of natural compounds including bufalin and digoxin widely used in the treatment of heart failure, and more recently have exhibited anticancer effects *in vitro* and *in vivo* via inhibition of the sodium pump [5]. They are known to be ligands for sodium pump which is overexpressed in many cancers promising a drug target in cancers [5, 7]. Several phase I and phase II clinical trials with cardiac glycosides such as digoxin, Anvirzel, and huachansu, either alone or more often in combination with other anticancer agents, have shown acceptable safety profiles [8]. Our previous studies have shown that cardiac glycosides induce a delay mitotic entry in many cancer cells [9], but the underlying mechanisms have not been completely understood.

Recently, crystal structures indicated that cardiac glycosides bufalin and digoxin have high affinity to sodium pump in the phosphoenzyme (E2P) form and block the extracellular cation exchange, which result in inhibitory effect on the sodium pump [10]. Recent studies have shown that cardiac glycosides including bufalin at nanomolar concentrations induce cell cycle arrest, apoptosis, autophagy, or inhibition of invasion and migration via inhibition of phosphoinositide 3-kinase (PI3K)/protein kinase B (Akt)/the mammalian target of rapamycin (mTOR) pathway, STAT3, NF-κB , or HIF1α, induction of ROS accumulation, or activation of amitogen-activated protein kinase (MAPK) ERK cascade [9, 11–14].

Mitosis transition is positively regulated by mitotic kinase, including Aurora kinases and Plk1, which are required for mitotic entry, spindle formation, chromosome segregation and cytokinesis [15]. To date, signal pathways for Aurora kinases and Plk1 in cardiac glycosides killing cancer cells are still poorly understood. Plk1 localizes at both centrosomes and kinetochores [16]. Aurora A has been reported to recruit to mitotic centrosomes and Aurora B to unattached kinetochores mediated by Plk1. Plk1 depletion or inhibition blocks Aurora A localization at centrosomes and impairs centrosome maturation [17]. Inhibition of Plk1 kinase activity prevents Aurora B activation [18]. Aurora A and B are frequently overexpressed in many cancers including colon, cervix, breast, lung, pancreas, and liver [19]. Aurora A disruption causes failure of mitotic exit. Inhibition of Aurora B with hesperadin leads to polyploid nuclei accumulation, decondensation of misaligned chromosomes, and followed by mitotic exit without cytokinesis. It is still unclear how mitotic kinases such as Aurora A and Aurora B are regulated during G2/M phase progression.

In this study, cell cycle progression in synchronized cells after cardiac glycoside bufalin treatment has been analyzed by using RNA interference techniques and pharmacological methods. Our data indicate that bufalin induces a delay of mitotic entry via inhibition of PI3K/Akt-dependent Aurora A/B activation, indicating the potential importance of bufalin for treatment of cancers. This finding has filled in a lot of gaps in current understanding of the molecular mechanism involved in cardiac glycosides-mediated mitotic arrest.

## RESULTS

### Bufalin treatment leads to a delay of mitotic entry and then mitotic arrest

**Figure 1:**
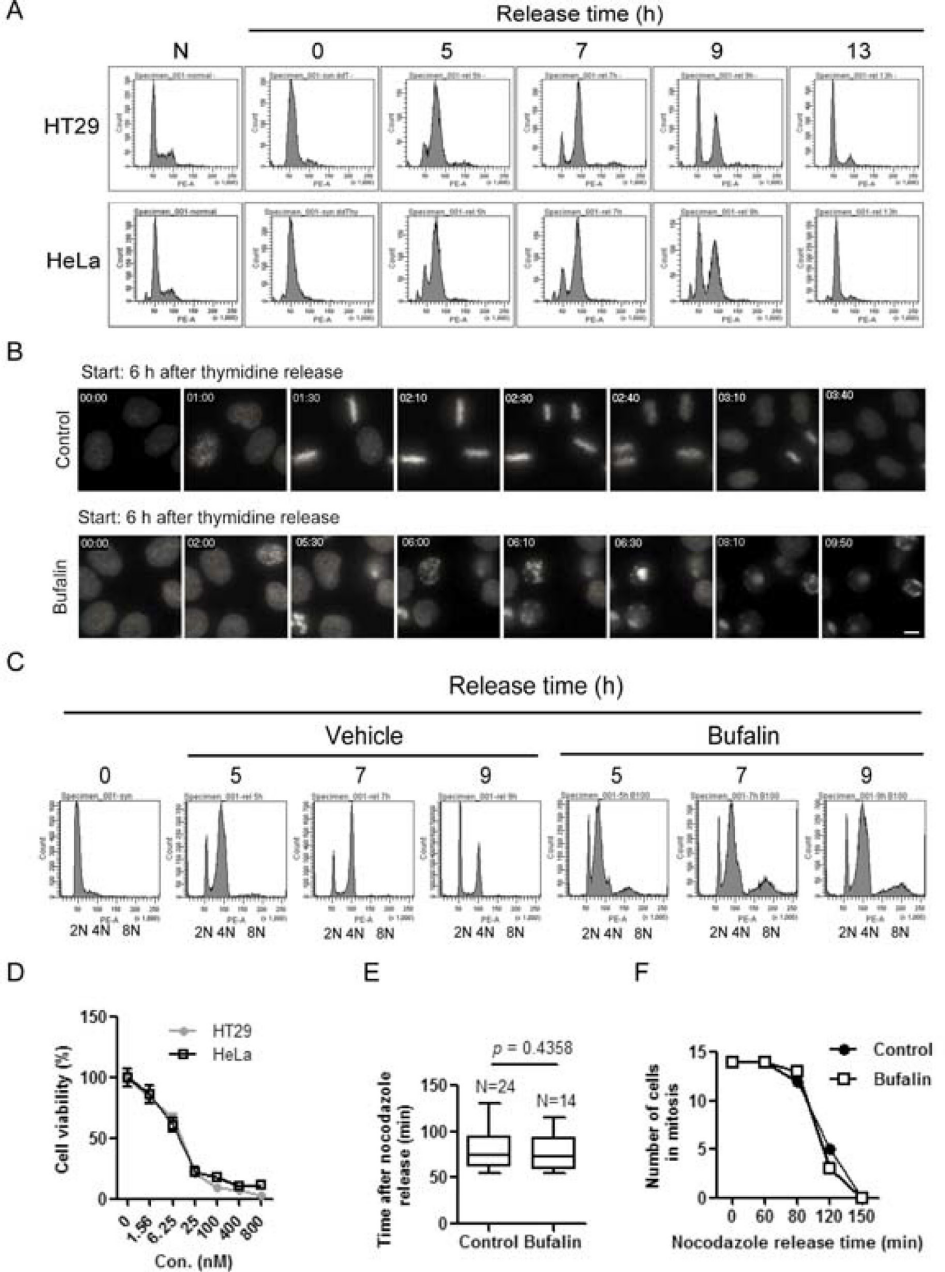
Bufalin treatment lends to a delay of mitotic entry and then mitotic arrest (**A**) HT-29 and HeLa cells were synchronized by a double thymidine block and then released into fresh 10% FBS medium for different time intervals. Cells were stained with PI (propidium iodide) and followed by analysis with a flow cytometer. (**B**) HeLa cells stably expressing H2B-YFP were blocked at the G1-S boundary by double thymidine treatment. Images were started 6 h after cells at the time of release from thymidine block to vehicle (0.1% DMSO) or 100 nM bufalin. Selected images containing the typical behavior of the population (e.g. start of recording, mitotic entry, mitotic exit or apoptosis, at the end of experiment) from time-lapse movies are shown. Time is displayed in hh:mm. The scale bar indicates 10 μm. (**C**) HT-29 cells were synchronized by thymidine and then released for different time intervals in the presence or absence of 100 nM bufalin. Cells were stained with PI and analyzed by flow cytometry. (**D**) HT-29 and HeLa cells were exposed to different concentrations of bufalin for 48 h and then incubated with 5 μg/ml MTT for 3 h. The cell viability was analyzed by MTT assay. Data shown represent the means ± SEM of three independent experiments. (**E**) Nocodazole-synchronized HeLa cells stably expressing H2B-YFP were released into 10% FBS medium for 20 min and then exposed to 100 nM bufalin. At 40 min, cells were filmed each 5 min under an Olympus microscope. The bars indicate the time spent in cell division from chromosome condensation to chromosome division. Data shown represent the means ± SEM of at least five independent experiments. (**F**) Quantification of number of cells in mitosis at each time points for the experiment described in (E). *n* = 14 for each group from three independent experiments.

To clearly demonstrate the cell cycle progression, cells were released for different time intervals from double thymidine block. The cell-cycle distribution was analyzed by flow cytometry. As shown in Figure 1A, double thymidine treatment caused cells arrest at G1-S phase. After release from a double thymidine block, cells started to enter S phase at 5 h and then G2/M phase at 7 h, followed by nearly completely passing through M phase at 13 h. Therefore, the G1-S boundary cells of HeLa cells stably expressing histone H2B-YFP was released from a double thymidine block. At 6 h, the effect of bufalin on G2/M phase was started to track under a time-lapse microscope. Control cells without bufalin treatment could succeed in going through G2/M phase, as indicated by the presence of sister chromatid condensation, chromosome alignment and segregation (Figure 1B), while bufalin-treated cells delayed mitotic entry followed by mitotic arrest, as characterized by the observed chromatid condensation and failure of chromosomes alignment and segregation (Figure 1B). In order to confirm this finding, cell cycle progression was analyzed in thymidine-synchronized HT-29 cells in the presence or absence of bufalin. As shown in Figure 1C, most of control cells succeeded in going through mitosis at 9 h, while bufalin treated cells were significantly arrested at G2/M phase with 4N DNA, some of which with 8N DNA. Cell viability data further demonstrated that bufalin reduces cancer cells proliferation (Figure 1D). Regarding the timing of mitosis, from early prophase entry to anaphase completion, there is no significant difference between bufalin-treated cells and control cells (Figure 1E). In addition, after release from a nocodazole block, the number of bufalin-treated cells in mitosis at different time points is similar to control, indicating bufalin does not block cytokenesis (Figure 1F). Taken together, bufalin treatment induces a delay of mitotic entry and then mitotic arrest.

### Bufalin reduces mitotic marker histone H3 phosphorylation

**Figure 2:**
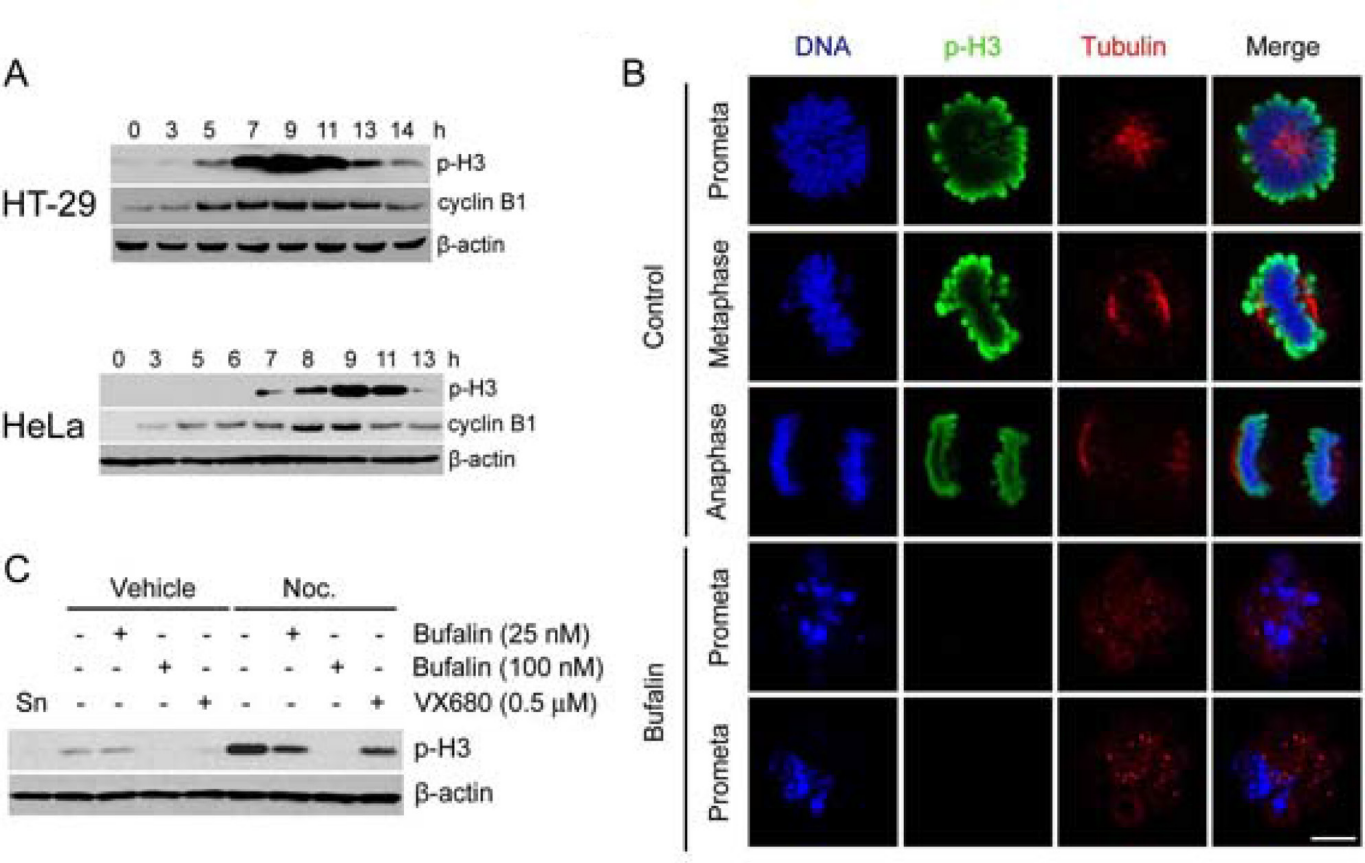
Bufalin reduces mitotic marker histone H3 phosphorylation (**A**) Western blot analysis of mitotic index phospho-histone H3 (Ser10) (p-H3) and cyclin B1 in thymidine-synchronized HT-29 and HeLa cells after release in 10% FBS medium for different time intervals. (**B**) Thymidine-synchronized HeLa cells were released in the presence or absence of bufalin (100 nM) for 9 h and followed by fixation with 70% ethanol and permeabilization with 0.1% Triton-X 100. The cells were stained with phospho-histone H3 (Ser10) (p-H3) and taken images under a confocal microscope. (**C**) Western blot analysis of mitotic index phospho-histone H3 (Ser10) (p-H3) in thymidine-synchronized HeLa cells 13 h after release in the presence of bufalin (25, 100 nM), Nocodazole (Noc. 0.33 μM), VX680 (0.5 μM), a combination of two of three drugs, or equal amount of DMSO. Abbreviation is as follow: Sn, synchronized cells.

Because bufalin treatment induced a delay of mitotic entry and mitotic arrest, we asked whether bufalin regulated mitotic marker. A mitotic marker namely phosphorylation of histone H3 at serine 10 is upregulated in late G2 and M phase for chromosome condensation [20]. In this study, we analyzed the protein levels of histone H3 at Ser 10 phosphorylation (p-H3) as well as cyclin B1 by Western blot. Cyclin B1 was used to track G2/M phase. Phosphorylation of histone H3 started to increase at 5 h for HT-29 cells and 7 h for HeLa cells, and then dramatically decreased at 13 h in both cells after release from a double thymidine block (Figure 2A). This finding is consistent with cyclin B1. Therefore, we detected the p-H3 signal by immunofluorescence staining in thymidine-synchronized HeLa cells after 9 h of release into vehicle (0.1% DMSO) or bufalin. As shown in Figure 2B, the phosphorylation of histone H3 was completely reduced, accompanied by failure of chromosome alignment after bufalin treatment. In order to further confirm this finding, the p-H3 levels were analyzed in thymidine-synchronized cells after 13 h release into bufalin, nocodazole, Aurora A/B inhibitor VX680, or a combination of two of these three drugs. The p-H3 levels were significantly reduced in bufalin and nocodazole, VX680 and nocodazole cotreatment cells compared with nocodazole treatment only, suggesting bufalin reduces histone H3 phosphorylation in a way similar to VX680 (Figure 2C). Taken together, these results indicate that bufalin reduces the mitotic marker histone H3 at Ser10 phosphorylation.

### Bufalin downregulates Aurora A and B in protein and phosphorylation levels

**Figure 3:**
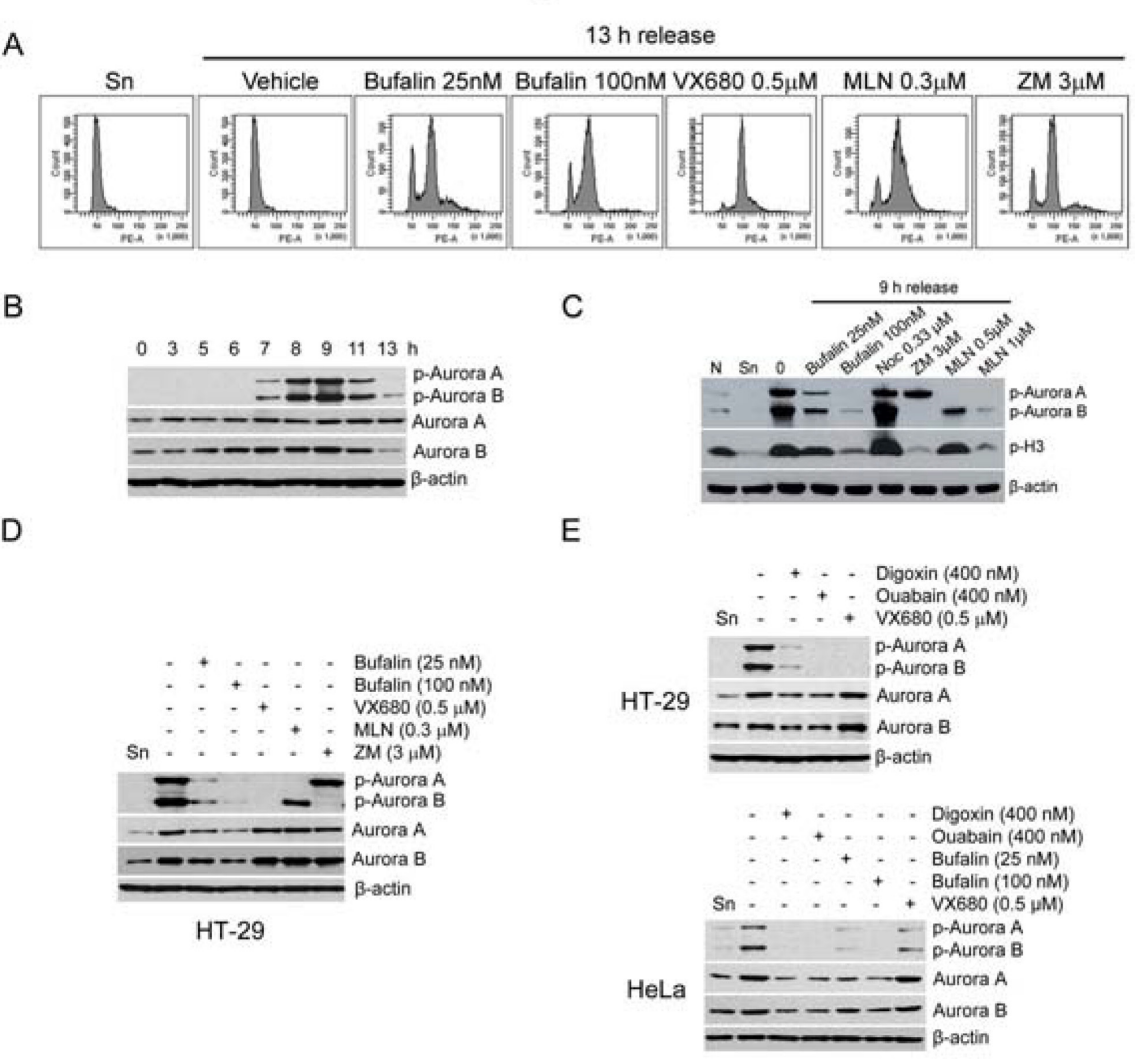
Bufalin downregulates Aurora A and B in protein and phosphorylation levels (**A**) HT-29 cells were synchronized to the G1-S boundary by double thymidine arrest and then exposed to Aurora A/B inhibitor VX680 (0.5 μM), Aurora A inhibitor MLN8237 (MLN; 0.3 μM), or Aurora B inhibitor ZM447439 (ZM; 3 μM) for 13 h. Cells were analyzed by flow cytometry. (**B**) Thymidine synchronized HT-29 cells were released into 10% FBS medium for indicated time intervals. The phospho-Aurora A (Thr288)/Aurora B (Thr232) and Aurora A/B were analyzed by Western blot. (**C**–**D**) Effect of bufalin on phospho-Aurora A (Thr288)/Aurora B (Thr232) and Aurora A/B in HT-29 cells. HT-29 cells were synchronized by double thymidine treatment and then treated with bufalin (100 nM), VX680 (0.5 μM), MLN8237 (MLN; 0.3 μM), or ZM447439 (ZM; 3 μM) for 9 h. The total lysates of cells were blotted for phospho-Aurora A (Thr288) and B (Thr232), p-H3, Aurora A/B, and β-actin (loading control). Abbreviation is as follow: Sn, synchronized cells. (**E**) Western blot analysis of phospho-Aurora A (Thr288)/Aurora B (Thr232) and Aurora A/B in HT-29 and HeLa cells after other sodium pump inhibitors digoxin and ouabain treatment. HT-29 or HeLa cells were synchronized by double thymidine arrest and released into the 10% FBS medium with bufalin (25, 100 nM), digoxin (400 nM), ouabain (400 nM), or VX680 (0.5 μM) for 9 h.

As shown in Figures 2B and 2C, bufalin reduced the phosphorylation of histone H3 at serine 10. We guessed that bufalin might inhibit the activity of Aurora B which is required for histone H3 phosphorylation [21]. Aurora A/B are required for both centrosome separation and spindle assembly. Therefore, we firstly analyzed whether bufalin induced mitotic arrest similar to Aurora A/B inhibitor VX680, Aurora A inhibitor MLN8237, or Aurora B inhibitor ZM447439. As shown in Figure 3A, bufalin could function like VX680, MLN8237, and ZM447439 to arrest cell in mitosis. In order to detect the effect of bufalin on Aurora A/B, we evaluated the total protein as well as active form of Aurora A/B in thymidine-synchronized cells after release for different time intervals. The active form of Aurora A (phospho-Aurora A at Thr288) and Aurora B (phospho-Aurora B at Thr232) reached the highest expression levels at 9 h and then decreased at 13 h after release from double thymidine block (Figure 3B). Therefore, we detected the protein levels of phospho-Aurora A (Thr288), phospho-Aurora B (Thr232), as well as p-H3 (Ser10) in HT-29 cells 9 h after release from double thymidine block and exposure to bufalin and Aurora A/B inhibitors. As shown in Figure 3C, bufalin significantly reduced both phospho-Aurora A (Thr288) and phospho-Aurora B (Thr232). Also, bufalin reduced p-H3 (Ser10) that is attributed by inhibition of Aurora B, similar to the action of Aurora B inhibitor ZM447439. In order to further confirm this finding, we detected other cardiac glycosides on total protein as well as active form of Aurora A/B. As shown in Figure 3D–3E, bufalin and other sodium pump inhibitors (e.g. digoxin and ouabain) significantly reduced the phosphorylation of Aurora A/B in both HT-29 and HeLa cells in a way similar to VX680. In addition, total protein levels of Aurora A/B in sodium pump inhibitors (e.g. bufalin, digoxin, ouabain) treated HT-29 and HeLa cells were also partly reduced (Figure 3D–3E). Taken together, these results suggest that bufalin-caused mitotic entry delay and mitotic arrest are attributed to downregulation of total protein as well as active form of Aurora A and B.

### Bufalin prevents Aurora A recruitment to mitotic centrosomes and Aurora B recruitment to unattached kinetochores

**Figure 4:**
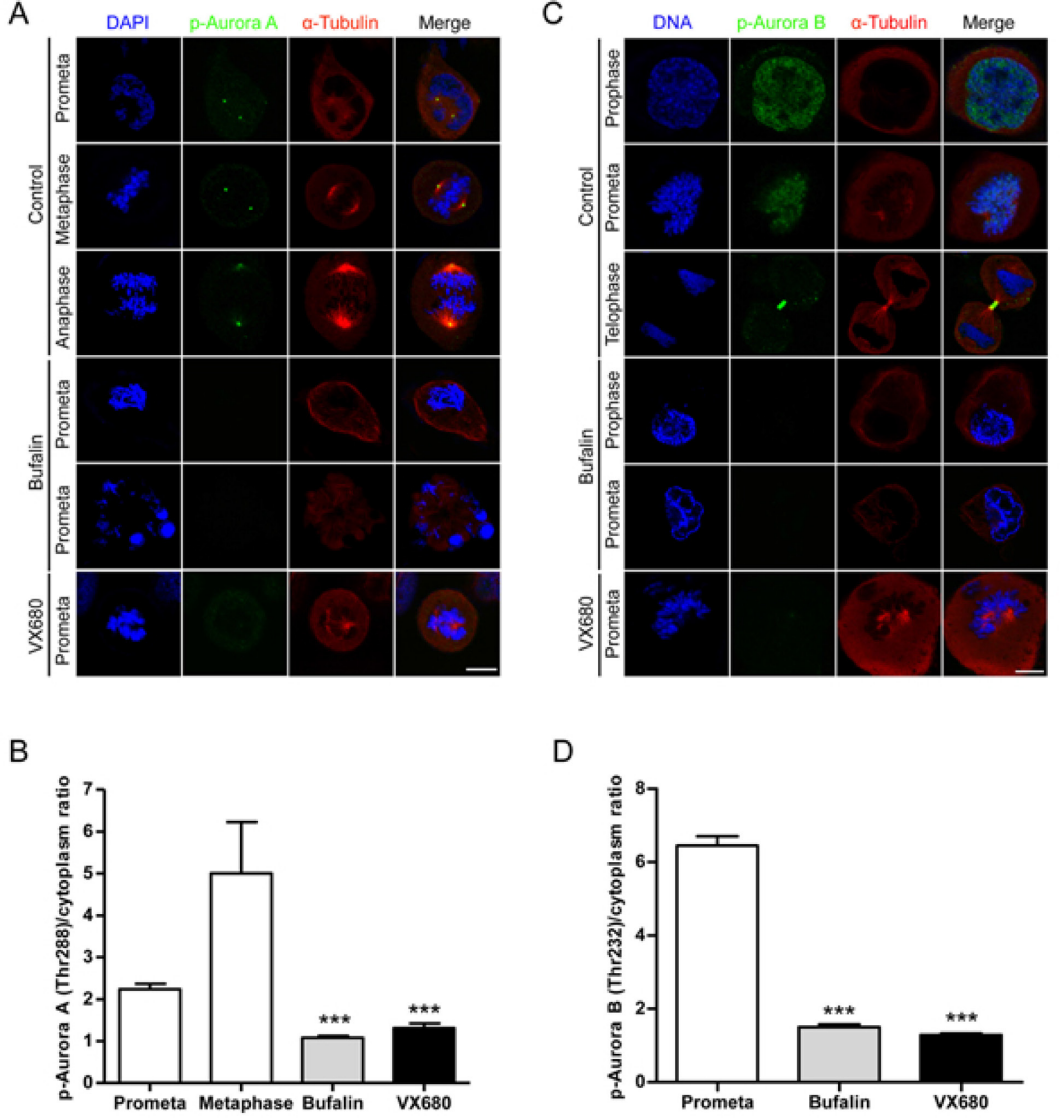
Bufalin prevents Aurora A recruitment to mitotic centrosomes and Aurora B recruitment to unattached kinetochores (**A**) HeLa cells were synchronized by a single thymidine treatment, released in the presence or absence of bufalin (100 nM) for 9 h, and stained for phospho-Aurora A (Green), α-tubulin (Red) and DNA (Blue). The scale bar represents 10 μm. (**B**) The phospho-Aurora A (Thr288) staining signals in (A) were normalized to the intensity in a same-size cytoplasmic region for at least five prometaphase cells per condition from three different experiments. ^***^p < 0.001, versus control prometaphase. Error bar represents SEM. (**C**) Thymidine-synchronized HeLa cells were treated with or without bufalin (100 nM) for 9 h and then stained for phospho-Aurora B (Green), α-tubulin (Red) and DNA (Blue). The scale bar represents 10 μm. (**D**) For quantification of the intensity of phospho-Aurora B (Thr232) in (C), more than 88 phospho-Aurora B (Thr232) staining signals from at least five prometaphase cells were analyzed each in control, bufalin (100 nM) and VX680 (0.5 μM) arrest. ^***^p < 0.001, versus control prometaphase. Error bar represents SEM.

In order to evaluate the role of Aurora A/B in bufalin-caused mitotic arrest, we analyzed the distribution of the active form of Aurora A/B in bufalin-treated cells by immunofluorescence staining. In control cells, we could clearly observe phospho-Aurora A (Thr288) brightly stained from prophase to anaphase that was fully or partially attached with microtubules (Figure 4A). Previous studies reported that Aurora A was essential for attachment between centrosomes and microtubules [22]. In contrast, after bufalin treatment, more than 90% of cells had low or no phospho-Aurora A stained signals and did not enter metaphase. By quantification of these signals, it showed that the intensity of staining for phospho-Aurora A at centrosomes in mitotic cells was significantly reduced by bufalin (Figure 4B). The phosphorylation of Aurora B at Thr232 is required for kinetochore-microtubule attachment during mitotic progression [23]. We therefore analyzed the distribution of phospho-Aurora B (Thr232) in bufalin-treated HeLa cells. As shown in Figure 4C, control prometaphase cells had brightly stained signals. These signals represent phospho-Aurora B that was fully accumulated at kinetochores to control microtubule-kinetochore attachment. However, bufalin-arrested prometaphase cells exhibiting a weakly stained phospho-Aurora B signals were observed compared with control cells. The intensity of phospho-Aurora B signals was reduced more than 4-fold that of its intensity at kinetochores in control metaphase cells (Figure 4D). As shown in Figure 4A and 4C, it was found that chromosomes in bufalin-arrested mitotic cells condensed normally, but failed to align at the metaphase plate. This result is similar to the phenotypic changes in Plk1 inhibitor and Aurora A/B inhibitor VX680 treated cells with chromosome condensation and failure to alignment [24, 25]. Plk1 controls the localization of Aurora A to centrosomes and of Aurora B to unattached kinetochores. Our previous work indicated that Plk1 was involved in bufalin-induced mitotic arrest [9]. Here, we found that Aurora A and B are also involved in this action. Taken together, bufalin prevents Aurora A recruitment to mitotic centrosomes and Aurora B recruitment to unattached kinetochores via inhibition of activation of Aurora A and Aurora B.

### Bufalin regulates the activities of Aurora A/B through PI3K-Akt-dependent pathway

**Figure 5:**
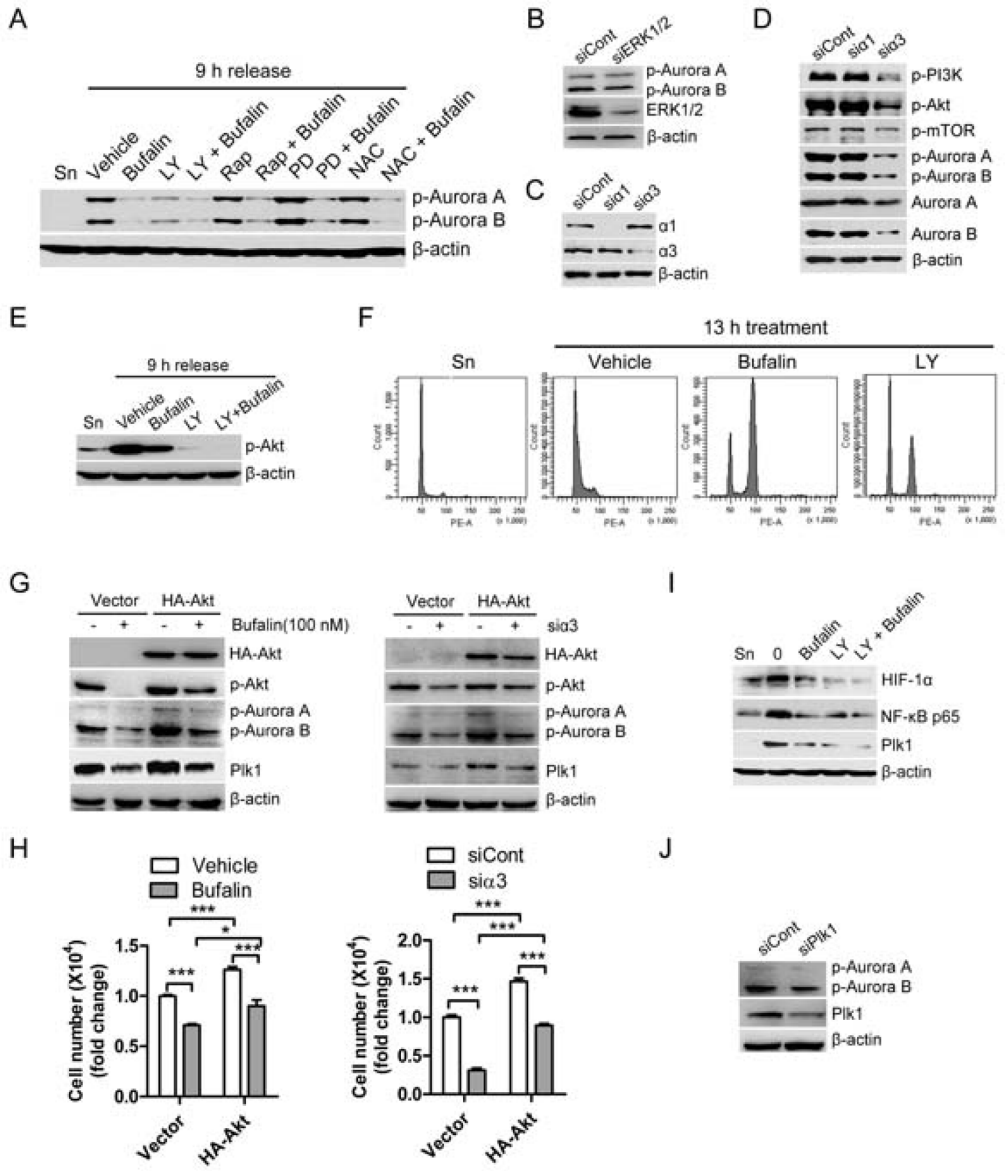
Bufalin regulates the activities of Aurora A/B through PI3K-Akt-dependent pathway (**A**) Thymidine synchronized HT-29 cells were released for 9 h in the presence of vehicle (0.1% DMSO), bufalin (100 nM), LY249002 (LY; 20 μM), rapamycin (Rap; 200 nM), PD98059 (PD; 20 μM), or NAC (10 mM). The phospho-Aurora A (Thr288)/Aurora B (Thr232) and β-actin were analyzed by Western blot. (**B**) HT-29 cells were transfected with Erk1/2 siRNA for 33 h, exposed to 2 mM thymidine for 15 h and followed by harvest after 9 h of release into 10% FBS medium. The protein levels of p-Aurora A/B were analyzed by Western blot. (**C**–**D**) HT-29 cells were transfected with siRNAs against sodium pump α1 and α3 for 33 h, exposed to 2 mM thymidine for 15 h and followed by harvest after 9 h of release into 10% FBS medium. (C) The knockdown efficiency of α1 siRNA and α3 siRNA was analyzed by Western blot. (D) Effect of sodium pump on the activities of PI3K, Akt, mTOR, and Aurora A/B was analyzed by Western blot. (**E**–**F**) Before entering into mitosis, thymidine-synchronized HT-29 cells were released for 5 h and then exposed to bufalin (100 nM), LY294002 (LY; 100 μM), a combination of two drugs, or equal amount of DMSO for 4 h. (E) Effect of PI3K inhibitor LY294002 on active form of Akt. (F) Effect of PI3K pathway on cell progression. (**G**–**H**) HT-29 cells were transfected with empty vector or HA-Akt for 24 h and then treated with bufalin for 24 h, or cotransfected with HA-Akt plus α3 siRNA for 48 h. (H) Cell number was counted for each group. (G) The protein levels of HA-Akt, p-Akt, p-Aurora A (Thr288)/B (Thr232), Plk1 and β-actin were analyzed by Western blot. (**I**) Before entering into mitosis, thymidine-synchronized HT-29 cells were released for 5 h and then exposed to bufalin (100 nM), LY294002 (LY; 100 μM), a combination of two drugs, or equal amount of DMSO for 4 h. The protein levels of NF-κB p65, HIF1α and Plk1 were analyzed by Western blot. (**J**) HT-29 cells were transfected with Plk1 siRNA for 33 h, exposed to 2 mM thymidine for 15 h and followed by harvest after 9 h of release into 10% FBS medium.

Recent studies reported that cardiac glycosides-induced cell cycle arrest and cell death are closely associated with the upregulation of ROS production as well as the inhibition of PI3K/Akt, mTOR, and ERK [11, 13]. We thus tested whether those pathways are involved in regulation of active form of Aurora A/B. As shown in Figure 5A, PI3K inhibitor LY249002, but not mTOR inhibitor rapamycin, MEK inhibitor PD98059 or ROS scavenger NAC, was able to significantly inhibit of active form of Aurora A/B. Consistent with PD98059 effect, silencing of ERK1/2 by siRNA also did not dramatically reduce phosphorylation of Aurora A/B (Figure 5B). These findings indicate that PI3K is closely associated to regulation of Aurora A/B. In order to confirm that silencing of sodium pump also affects this pathway, we firstly detected the efficiency of silencing of sodium pump by using RNA interference in HT-29 cells (Figure 5C). Consistent with previous action of cardiac glycosides, our results showed that silencing of sodium pump catalytic subunit α3 but not α1 significantly reduced protein levels as well as active form of Aurora A/B (Figure 5D). This finding is similar to our previous report that α3 plays a critical role in sodium pump-mediated cell proliferation of colorectal cancer. In addition, silencing of sodium pump dramatically inhibited both PI3K and Akt but not mTOR in human colon cancer cells (Figure 5D). In order to confirm this finding, we tested the effect of bufalin, PI3K inhibitor LY294002, or a combination of these two drugs on active form of Akt. As shown in Figure 5E, bufalin could significantly inhibit the phosphorylation of Akt and LY294002 further enhanced this action. Furthermore, LY294002 blocked cells at G2/M phase like bufalin (Figure 5F). In order to further confirm the effect of PI3K/Akt on Aurora A/B activity, we tested if overexpression of Akt could reverse the inhibition of Aurora A/B activity as well as cell proliferation mediated by both bufalin treatment and silencing of sodium pump α3. As shown in Figure 5G and 5H, both bufalin treatment and silencing of α3 mediated the inhibition of Aurora A/B activity and cell proliferation were partly rescued by overexpression of Akt. In addition, both bufalin and silencing of α3 mediated downregulation of Plk1 was also reversed by the overexpression of Akt (Figure 5G). PI3K inhibitor LY249002 similar to bufalin could induce the downregulation of NF-κB p65, HIF1α and Plk1 (Figure 5I). Our previous study demonstrated that buflain downregulated Plk1 via NF-κB and HIF1α [9]. These findings indicate that PI3K-Akt pathway downregulates Plk1 through NF-κB and HIF1α. Silencing of Plk1 did not significantly reduce active form of Aurora A/B in human colon cancer cells, suggesting Aurora A/B and Plk1 work in parallel during bufalin-induced mitotic arrest (Figure 5J). Taken together, PI3K-Akt pathway is closely involved in bufalin-induced mitotic arrest via inhibition of Aurora A/B activation and downregulation of Plk1.

## DISCUSSION AND CONCLUSIONS

Our previous studies have demonstrated that sodium pump inhibitors cardiac glycosides (e.g. bufalin, digoxin and ouabain) can delay mitotic entry and then arrest cell cycle in mitosis in a lot of cancer cells via Plk1 pathway [9]. However, the underlying mechanism of this action remains poorly understood. In this study, we have used time-lapse experiments, Western blot, as well as immunofluorescence staining in the synchronized cells to gain novel insights into the molecular mechanism involved in cardiac glycosides-mediated mitotic entry delay and mitotic arrest.

During the past decades, the phosphorylation of histone H3 at serine 10 has been clearly demonstrated to control the initiation of chromosome condensation in mitosis and meiosis in a wide range of organisms from worms to animals, including human. Gurley *et al.* reported that histone H3 was phosphorylated to stimulate chromosome condensation in mitosis [26], which is consistent with our finding that mitotic cells had high levels of phospho-histone H3. However, Hsu *et al.* reported that phospho-histone H3 serine 10 is not necessary for proper chromosome dynamics in yeast, as characterized by loss of H3 phosphorylation could not completely inhibit chromosome condensation [27]. This finding is not consistent with the phenotypic changes observed in other eukaryotic systems examined to date. One reasonable cause for this difference is that other histones such as histone H2B, which is modified by the upstream histone H3 of Aurora/Ipl1p kinase, may rescue the functional loss of Ser 10 phosphorylation of histone H3 in yeast. Giet and Glover found that drosophila Aurora B kinase was essential for histone H3 phosphorylation at Ser10 and the decrease of the phosphorylation of histone H3 by knockdown of aurora B was shown to cause the failure of chromosome condensation and spindle formation [21]. Consistent with this finding, bufalin-treated cells with no or low levels of phospho-histone H3 (Ser10) and phospho-Aurora B (Thr232) failed to organize the spindle formation.

Time-lapse video can monitor changes in cell cycle kinetics of cells and can directly show the interphase and mitotic duration in cells. As histone H2B is the conserved and stable protein in eukaryotic cells [28], it has been widely used to study chromosomal division in living cells by labeling with fluorescent reporter proteins, such as GFP or YFP. In our study, we found that bufalin-treated cells delayed entry into mitosis and then failed to pass through mitosis, followed by apoptosis. The Aurora B inhibitor hesperidin could induce cells exit mitosis without cytokinesis with polyploid nuclei, and misaligned chromosomes decondensation. Disruption of Aurora A causes cells chromosome condensation but failure to pass through mitosis [29]. Inhibition of Plk1 causes mitotic arrest with chromosome condensation and failure to alignment, followed by mitotic arrest for a long period of time or apoptosis. Morphology of chromosome condensation and monopolar spindles in Aurora A/B inhibitor VX-680 treated cells was similar to that in Aurora A deprived cells. Our previous study indicated that sodium pump inhibitor bufalin reduced Plk1 protein levels. In this study, bufalin reduced protein levels as well as active form of mitotic kinase Aurora A/B, and arrested cells in mitosis with chromosome condensation and failure of chromosome alignment. These findings indicated that Aurora A/B together with Plk1 are closely involved in cardiac glycosides-induced a delay of mitotic entry and then mitotic arrest. As Plk1 is required for the localization of Aurora A/B, the decrease of Plk1 level by bufalin made the phenotype similar to Plk1 inhibition. Previous study reported that Plk1 was required for Aurora B activation by phosphorylation at Ser20, which is essential for accurate chromosome segregation and faithful completion of cytokinesis [18]. Plk1 is first phosphorylated on Thr210 in G2 phase by the kinase Aurora A, in concert with its cofactor Bora. Bora/aurora-A-dependent phosphorylation is a prerequisite for Plk1 to promote mitotic entry [30, 31]. These studies indicated that Plk1 and Aurora A/B regulate each other at phosphorylation levels. In our study, bufalin reduced Plk1 in both mRNA and protein levels and Aurora A/B in phosphorylation levels. Silencing of Plk1 did not significantly reduce the phosphorylation of Aurora A/B. These results indicate Aurora A/B and Plk1 work in parallel in bufalin-induced mitotic arrest.

PI3K pathway plays an important role in regulating cell survival, angiogenesis, cell invasion, and metastasis [32]. Liu *et al.* reported that integrin α2/FAK/AKT1/ GSK3β signaling was involved in cell cycle regulation of bufalin in cervical cancer [33]. However, the link between PI3K and Aurora kinase has been limitedly described as well as the mechanism by which PI3K regulates mitotic kinase is still poorly understood. Liu *et al.* showed that PI3K inhibitor LY294002 significantly downregulated the protein levels of Aurora A [34]. Lin *et al.* found that 16-Hydroxycleroda-3,13-dien-15,16-olide (PL3) induced G2/M phase arrest through PI3K-mediated deregulation of the protein levels of Aurora B [35]. In our study, we found that bufalin dramatically reduced the phosphorylation rather protein levels of Aurora A/B. The effect of LY294002 similar to bufalin significantly reduced phosphorylation of Aurora A/B. Interestingly, recent studies showed that knockdown of Aurora B suppressed lung and osteosarcoma cell invasion and migration via inhibiting PI3K-Akt pathway [36, 37]. ERK was reported to reduce Aurora A/B in melanoma cells [37, 38]. However, silencing of ERK significantly did not reduce active form of Aurora A/B in colon cancer cells. Our previous study reported that MEK/ERK inhibitor PD98059 could not reverse bufalin-mediated inhibition of cell proliferation in human colon cancer cells [39]. In addition, ERK and ROS levels have been reported to involve in bufalin-mediated cell cycle arrest and cell death [40, 41]. In our study, ERK inhibitor PD98059 and ROS scavenger NAC did not regulate phosphorylation of Aurora A/B, indicating ERK and ROS pathways are not involved in bufalin-induced mitotic arrest.

In this study, we found that sodium pump inhibitor bufalin-mediated mitotic entry delay and mitotic arrest were attributed to the reduction of activation of Aurora A/B through PI3K-Akt pathway, indicating that sodium pump results in activation of Aurora A/B through PI3K-Akt pathway, which finally leads to cancer proliferation. Our results not only enrich our understanding of the action of cardiac glycoside bufalin on anticancer growth, but also give our novel insights into the implication of sodium pump for cancer treatment.

## MATERIALS AND METHODS

### Reagents and antibodies

Digoxin (D6003), ouabain (O3125), McCoy’s 5A medium (M4892), and anti-α-tubulin antibody (T6074) were purchased from Sigma. Bufalin (025-15241) was obtained from Wako Pure Chemical Industries. LY294002 (9901), anti-Aurora A/AIK (1G4) (4718), Anti-phospho-Aurora A (Thr288)/Aurora B (Thr232) (2914), anti-phospho-Aurora A (Thr 288) (3079), and anti-histone H3 (9715) were obtained from Cell Signaling Technology. The anti-histone H3 (phospho S10) (ab14955) antibody was from Abcam. Anti-Aurora B antibody (1788-1) was from Epitomics Inc. Anti-Aurora B pT232 (600-401-677) and anti-α-Tubulin (600-301-880) antibodies were obtained from Rockland immunochemicals. Alexa Fluor 488 Donkey anti-Rabbit lgG (A21206) and Alexa Fluor 555 Donkey anti-Mouse lgG (A31570) antibodies were purchased from Life Technologies Corporation. PI (propidium iodide)/RNase staining buffer solution (550825) was purchased from BD Pharmingen. Fetal bovine serum (FBS; 16000-044) and Dulbecco’s Modified Eagle medium (DMEM; 12800-017) were gotten from Gibco Invitrogen. Anti-mouse lgG-HRP (*sc-2005*) and anti-rabbit lgG-HRP (*sc-2004*) antibodies were obtained from Santa Cruz Biotechnology. Chemiluminescence HRP Substrate (WBKLS0500) antibody was from Millipore.

### Cell culture and synchronization

The human colon cancer HT-29 cells were cultured in McCoy’s 5A medium plus 10% FBS and the human cervical cancer HeLa cells in DMEM medium, respectively. For G2/M phase, cells were exposed to 0.33 μM nocodazole for 13 h. For G1-S boundary, cells were exposed to 2 mM thymidine for 17 h, washed with PBS for 3 times, released in fresh medium containing 10% FBS for 9 h and followed by exposure to 2 mM thymidine for 15 h.

### Cell cycle analysis

In order to evaluate the effect of bufalin on cell cycle progression, the bufalin or nocodazole treated cells were fixed overnight in ice cold 70% ethanol, washed with PBS and then exposed to PI/RNase A solution for 10 min. The percentage of cells in different phase was analyzed by flow cytometry.

### Time-lapse experiments

Images were taken under a Nikon fluorescence microscope or an Olympus FV1000 confocal microscope. Cells were incubated in a chamber maintained in humidified atmosphere containing 5% CO_2_ at 37^°^C during imaging. After 6 h of exposure to vehicle (0.1% DMSO) or 100 nM bufalin, the G1-S boundary cells were taken 1 frame every 10 or 15 min for 11 h under the microscope. For cytokinesis analysis, mitotic cells obtained after 13 h of exposure to 0.33 μM nocodazole were exposed to vehicle or bufalin for 20 min, and followed by capturing 1 frame every 5 min for 3 h. All images were processed by FV10-ASW1.6 (Olympus) and Adobe Photoshop 7.0 (Adobe Systems, San Jose, CA).

### Immunofluorescence staining

The procedure for immunofluorescence staining was described in a previous report [9]. Thymidine-synchronized cells grown on slides were fixed with 3.5% formaldehyde for 10 min, permeabilized with 0.1% Triton X-100 for 5 min, blocked with 1% BSA for 20 min, incubated with indicated primary antibodies for 1 h at room temperature, and followed by incubation with Alexa Fluor 488 and 555-conjugated secondary antibodies for 1 h. The slides were mounted with Vectashield mounting medium containing DAPI and images were taken. The staining intensity of images was measured by AlphaEaseFC software (Alpha Innotech Corporation, San Leandro, CA).

### siRNAs transfection and treatment

The siRNAs for α1 isoform of sodium pump (sense: 5′-GGGCAGUGUUUCAGGCUAA-3′, anti-sense: 5′-UU AGCCUGAAACACUGCCC-3′) was obtained from Genepharm. The α3 siRNA (sc-36012) for α3 isoform of sodium pump was obtained from Santa Cruz. The siRNAs for Plk1 (#6292) and Erk1/2 (#6560) were gotten from Cell Signaling Technology. A non-target siRNA (sense: 5′-UCUACGAGGCACGAGACUU-3′, and anti-sense: 5′-AACUCUCGUGCCUCGUAGA-3′) was used as a control. Cells were transfected with 75 nM control siRNA, 75 nM α1 siRNA, 37 nM α3 siRNA, 37 nM Plk1 siRNA, or 37 nM Erk1/2 siRNA using Lip2000 transfection reagent for 33 h, treated with 2 mM thymidine for 15 h and followed by release into fresh 10% FBS medium for 9 h. Total proteins were collected for Western blot.

### Western blot analysis

The procedure for Western blot analysis was described in a previous report [42]. The proteins were analyzed on a 10–12% SDS-PAGE and then transferred onto the nitrocellulose membrane. The membrane was incubated with the primary antibody respectively at 4^°^ C overnight, washed with TBST for 3 times, incubated with the horseradish peroxidase-conjugated secondary antibody. The appropriate bands were detected after exposure to the Chemiluminescence HRP Substrate.

### Statistical analysis

Statistical analysis was performed by a software from GraphPad Prism (GraphPad, San Diego, CA) using one-way analysis of variance (ANOVA) for comparison of more than two group. Data were expressed as mean ± SEM of more than 3 independent experiments. A *p* value < 0.05 was considered statistically significant.

## Abbreviations

H3: Histone H3
MLN: MLN8237
Noc: nocodazole
PI: propidium iodide
Prometa: prometaphase
ZM: ZM447439
Sn: synchronized cells
